# Intense, Passionate, Romantic Love: A Natural Addiction? How the Fields That Investigate Romance and Substance Abuse Can Inform Each Other

**DOI:** 10.3389/fpsyg.2016.00687

**Published:** 2016-05-10

**Authors:** Helen E. Fisher, Xiaomeng Xu, Arthur Aron, Lucy L. Brown

**Affiliations:** ^1^The Kinsey Institute, Indiana University, BloomingtonIN, USA; ^2^Department of Psychology, Idaho State University, PocatelloID, USA; ^3^Department of Psychology, The State University of New York Stony Brook, Stony BrookNY, USA; ^4^Department of Neurology, Albert Einstein College of Medicine, BronxNY, USA

**Keywords:** romantic love, addiction, ventral tegmental area, caudate

## Abstract

Individuals in the early stage of intense romantic love show many symptoms of substance and non-substance or behavioral addictions, including euphoria, craving, tolerance, emotional and physical dependence, withdrawal and relapse. We have proposed that romantic love is a natural (and often positive) addiction that evolved from mammalian antecedents by 4 million years ago as a survival mechanism to encourage hominin pair-bonding and reproduction, seen cross-culturally today in Homo sapiens. Brain scanning studies using functional magnetic resonance imaging support this view: feelings of intense romantic love engage regions of the brain’s “reward system,” specifically dopamine-rich regions, including the ventral tegmental area, also activated during drug and/or behavioral addiction. Thus, because the experience of romantic love shares reward pathways with a range of substance and behavioral addictions, it may influence the drug and/or behavioral addiction response. Indeed, a study of overnight abstinent smokers has shown that feelings of intense romantic love attenuate brain activity associated with cigarette cue-reactivity. Could socially rewarding experiences be therapeutic for drug and/or behavioral addictions? We suggest that “self expanding” experiences like romance and expanding one’s knowledge, experience and self-perception, may also affect drug and/or behavioral addiction behaviors. Further, because feelings of romantic love can progress into feelings of calm attachment, and because attachment engages more plastic forebrain regions, there is a rationale for therapies that may help substance and/or behavioral addiction by promoting activation of these forebrain systems through long-term, calm, positive attachments to others, including group therapies. Addiction is considered a negative (harmful) disorder that appears in a population subset; while romantic love is often a positive (as well as negative) state experienced by almost all humans. Thus, researchers have not categorized romantic love as a chemical or behavioral addiction. But by embracing data on romantic love, it’s classification as an evolved, natural, often positive but also powerfully negative addiction, and its neural similarity to many substance and non-substance addictive states, clinicians may develop more effective therapeutic approaches to alleviate a range of the addictions, including heartbreak–an almost universal human experience that can trigger stalking, clinical depression, suicide, homicide, and other crimes of passion.

## Introduction

We propose that romantic love is a natural addiction (Frascella et al., 2010) that evolved from mammalian antecedents (Fisher et al., 2006). Brain scanning studies show that feelings of intense romantic love engage regions of the brain’s “reward system,” specifically dopamine pathways associated with energy, focus, learning, motivation, ecstasy, and craving, including primary regions associated with substance addiction, such as the ventral tegmental area (VTA), caudate and accumbens (Breiter et al., 1997; Bartels and Zeki, 2000, 2004; Fisher et al., 2003, 2005, 2006, 2010; Aron et al., 2005; Ortigue et al., 2007; Acevedo et al., 2011; Xu et al., 2011). Several of these reward regions of the mesolimbic system associated with romantic love and substance addiction are also activated during non-substance or behavioral addiction, including viewing images of appealing food (Wang et al., 2004), shopping (Knutson et al., 2007), playing video games (Hoeft et al., 2008), and gambling (Breiter et al., 2001). Indeed, several researchers have taken the position that “addiction is a disease of the reward system” (Rosenberg and Feder, 2014). Moreover, men and women who are passionately in love and/or rejected in love show the basic symptoms of substance-related and gambling addiction listed in the Diagnostic and Statistical Manual of Mental Disorders-5, including craving, mood modification, tolerance, emotional and physical dependence and withdrawal. Relapse is also a common problem for those suffering with a substance and/or behavioral addiction, as well as among rejected lovers.

Because passionate romantic love is regularly associated with a suite of traits linked with all addictions, several psychologists have come to believe that romantic love can potentially become an addiction (Peele, 1975; Tennov, 1979; Hunter et al., 1981; Halpern, 1982; Schaef, 1989; Griffin-Shelley, 1991; Mellody et al., 1992). However, many define addiction as a pathological, problematic disorder (Reynaud et al., 2010); and because romantic love is a positive experience under many circumstances (i.e., not harmful), researchers remain hesitant to officially categorize romantic love as an addiction. But even when romantic love can’t be regarded as harmful, it is associated with intense craving and can impel the lover to believe, say and do dangerous and inappropriate things.

All forms of substance abuse, including alcohol, opioids, cocaine, amphetamines, cannabis, and tobacco activate reward pathways (Breiter et al., 1997; Melis et al., 2005; Volkow et al., 2007; Frascella et al., 2010; Koob and Volkow, 2010; Diana, 2013), as do several of the behavioral addictions (see Cuzen and Stein, 2014); and several of these same reward pathways are also found to be activated among men and women who are happily in love, as well as those rejected in love (Bartels and Zeki, 2000, 2004; Fisher et al., 2003, 2010; Aron et al., 2005; Ortigue et al., 2007; Acevedo et al., 2011; Xu et al., 2011). So regardless of its official diagnostic classification, we propose that romantic love should be considered as an addiction (Fisher, 2004, 2016): a positive addiction when one’s love is reciprocated, non-toxic and appropriate, and a negative addiction when one’s feelings of romantic love are socially inappropriate, toxic, not reciprocated and/or formally rejected (Fisher, 2004; Frascella et al., 2010).

Romantic love may have evolved at the basal radiation of the hominin clade some 4.4 million years ago in conjunction with the evolution of serial social monogamy and clandestine adultery–hallmarks of the human reproductive strategy (Fisher, 1998, 2004, 2011, 2016). Its purpose may have been to motivate our forebears to focus their mating time and energy on a single partner at a time, thus initiating the formation of a pair-bond to rear their young together as a team (Fisher, 1992, 1998, 2004, 2011, 2016; Fisher et al., 2006; Fletcher et al., 2015). Thus, as products of human evolution, the neural systems for romantic love and mate attachment could be considered as survival systems among humans.

## Addiction-Like Behaviors in Early Stage, Passionate Lovers: Euphoria, Obsession, Risky Behavior

Men and women in the early stage of intense passionate romantic love express many of the basic traits associated with all addiction (Tennov, 1979; Liebowitz, 1983; Hatfield and Sprecher, 1986; Harris, 1995; Lewis et al., 2000; Meloy and Fisher, 2005; American Psychiatric Association, 2013). Like all addicts, they focus on their beloved (salience); and they yearn for the beloved (craving). They feel a “rush” of exhilaration when seeing or thinking about him or her (euphoria/intoxication). As their relationship builds, the lover seeks to interact with the beloved more and more frequently (tolerance). If the beloved breaks off the relationship, the lover experiences the common signs of drug withdrawal, too, including protest, crying spells, lethargy, anxiety, insomnia, or hypersomnia, loss of appetite or binge eating, irritability and chronic loneliness. Like most addicts, rejected lovers also often go to extremes, even sometimes doing degrading or physically dangerous things to win back the beloved (Meloy, 1998; Lewis et al., 2000; Meloy and Fisher, 2005). Romantic partners are willing to sacrifice, even die for the other. Romantic jealousy is particularly dangerous and can lead to major crimes including homicide, and/or suicide. Lovers also relapse the way drug addicts do: long after the relationship is over, events, people, places, songs, and/or other external cues associated with their abandoning sweetheart can trigger memories and initiate renewed craving, obsessive thinking and/or compulsive calling, writing or showing up in hopes of rekindling the romance–despite what they suspect may lead to adverse consequences.

Passionate lovers also express strong sexual desire for the beloved; yet their yearning for emotional union tends to overshadow their craving for sexual union with him or her (Tennov, 1979). Most characteristic, the lover thinks obsessively about the beloved (intrusive thinking). Besotted lovers may also compulsively follow, incessantly call, write or unexpectedly appear, all in an effort to be with their beloved day and night (Tennov, 1979; Lewis et al., 2000; Meloy and Fisher, 2005). Paramount to this experience is intense motivation to win him or her. All these behaviors are common to those with substance addictions. However, not everyone exhibits these types of behaviors after a breakup, just as not everyone who uses a substance exhibits dependency and withdrawal effects (e.g., Shiffman, 1989; Shiffman et al., 1995; Shiffman and Paty, 2006; Haney, 2009).

## The Brain Systems Associated with Romantic Love

Neuroimaging studies of intense, passionate romantic love reveal the physiological underpinnings of this universal or near-universal human experience, and they all show activation of the VTA (Fisher et al., 2003, 2010; Bartels and Zeki, 2004; Aron et al., 2005; Ortigue et al., 2007; Zeki and Romaya, 2010; Acevedo et al., 2011; Xu et al., 2011). In our first experiment (Fisher et al., 2003; Aron et al., 2005), we used functional magnetic resonance imaging (fMRI) to study 10 women and 7 men who had recently fallen intensely and happily in love. All scored high on the Passionate Love Scale (Hatfield and Sprecher, 1986), a self-report questionnaire that measures the intensity of romantic feelings; all participants also reported that they spent more than 85% of their waking hours thinking of their beloved.

Participants alternately viewed a photograph of their sweetheart and a photograph of a familiar individual, interspersed with a distraction-attention task. Group activation occurred in several regions of the brain’s reward system, including the VTA and caudate nucleus (Fisher et al., 2003; Aron et al., 2005), regions associated with pleasure, general arousal, focused attention and motivation to pursue and acquire rewards and mediated primarily by dopamine system activity (Delgado et al., 2000; Schultz, 2000; Elliott et al., 2003). These regions of the reward system are directly associated with addiction in many studies of drugs of abuse (Breiter et al., 1997; Panksepp et al., 2002; Melis et al., 2005; Volkow et al., 2007; Frascella et al., 2010; Koob and Volkow, 2010; Diana, 2013) and behavioral addictions (see Cuzen and Stein, 2014).

These data from several studies indicate that individuals who are happily in the early stages of passionate love express activity in neural regions associated with drug and some behavioral addictions.

There is also a difference between “wanting” and “liking/pleasure” suggested by Berridge et al. (2009). As in substance addiction, “wanting” the romantic partner is different from “liking” a pretty face and finding pleasure in a beautiful sight. We found that brain activation to an attractive face (“liking”) was different from activation to the beloved partner (“wanting”): the former activated the left VTA, while the latter activated the right VTA (Aron et al., 2005). The result suggests the addictive aspects of romantic love are mediated through the right VTA, and that pleasure, or “liking” is different.

## Addiction-Like Behaviors Associated with Romantic Rejection: Craving, Relapse and Destructive Behavior

Cross-culturally, few men or women avoid suffering from romantic rejection at some point across their lives. In one American college community, 93% of both sexes queried reported that they had been spurned by someone whom they passionately loved; 95% reported they had rejected someone who was deeply in love with them (Baumeister et al., 1993). Romantic rejection can cause a profound sense of loss and negative affect (although this is not always the case e.g., Lewandowski and Bizzoco, 2007). Like many addictions, romantic rejection can also jeopardize one’s health, because abandonment rage stresses the heart, raises blood pressure and suppresses the immune system (Dozier, 2002). It can also induce clinical depression, and in extreme cases lead to suicide and/or homicide. Some broken-hearted lovers even die from heart attacks or strokes caused by their depression (Rosenthal, 2002). The suite of negative phenomena associated with rejection in love, including protest, the stress response, frustration attraction, abandonment rage, and jealousy, in conjunction with craving and withdrawal symptoms, most likely also contribute to the high worldwide incidence of crimes of passion (see Meloy, 1998; Meloy and Fisher, 2005).

One pathology is also regularly associated with romantic love, stalking. There are two common types of stalkers: those who sustain pursuit of a former sexual/romantic intimate who has rejected them; and those who pursue a stranger or acquaintance who has failed to return the stalker’s romantic overtures (Meloy and Fisher, 2005). In both cases, the stalker exhibits several of the characteristic components of all addictions, including focused attention on the love object, increased energy, following behaviors, and obsessive thinking about and impulsivity directed toward the victim, suggesting that stalking also activates aspects of the reward system in the brain (Meloy and Fisher, 2005) and may be akin to addiction. Another pathology, de Clerambault’s syndrome, also known as erotomania, has not been associated with addiction. This syndrome is characterized by the patient’s delusional notion that another person is madly in love with him or her; generally it is a young woman who believes that she is the love object of a man of higher social or professional standing. But because this syndrome has no direct association with reward system activity and may be a form of paranoid schizophrenia or other delusional disorder (Jordan and Howe, 1980; Kopelman et al., 2008) rather than addiction, discussion of this syndrome is beyond the scope of this paper.

It appears, however, as if evolution has overdone the negative response to romantic abandonment. But romantically rejected individuals have wasted precious courtship time and metabolic energy; they have lost essential economic and financial resources; their social alliances have been jeopardized; their daily rituals and habits have been altered; they may have lost property; and they have most likely experienced damage to their personal happiness, self-esteem and reputation (see Leary, 2001; Fisher, 2004). Most important, rejected lovers of reproductive age are likely to have lost breeding opportunities or a parenting partner for the offspring they have already produced—forms of reduced future genetic viability (Fisher, 2004). Thus, romantic rejection can have severe social, psychological, economic, and reproductive consequences.

## Romantic Rejection Also Activates Brain Regions Associated with Drug Craving

To identify some of the neural systems associated with this natural craving state elicited by romantic rejection, we used fMRI to study 10 women and 5 men who had recently been rejected by a partner, but reported that they were still intensely “in love” (Fisher et al., 2010). The average length of time since the initial rejection and the participants’ enrollment in the study was 63 days. All scored high on the Passionate Love Scale (Hatfield and Sprecher, 1986); all reported that they spent most of their waking hours thinking about the person who rejected them; and all yearned for their abandoning partner to return to the relationship.

Participants alternately viewed a photograph of their rejecting partner and a photograph of a familiar, emotionally neutral individual, interspersed with a distraction-attention task. Their responses while looking at their rejecter in the scanner included feelings of romantic passion, despair, joyous, and painful memories, rumination about why this had happened, and mental assessments of their gains and losses from the experience. Brain activations coupled with viewing the rejecter occurred in several regions of the brain’s reward system. Included were: the VTA associated with feelings of intense romantic love; the ventral pallidum associated with feelings of attachment; the insular cortex and the anterior cingulate associated with physical pain and the distress associated with physical pain; and the nucleus accumbens and orbitofrontal/prefrontal cortex associated with assessing one’s gains and losses, as well as craving and addiction (Fisher et al., 2010). Activity in several of these brain regions has been correlated with craving for cocaine and other drugs of abuse (Melis et al., 2005; Frascella et al., 2010; Koob and Volkow, 2010; Diana, 2013).

To understand the impact of right VTA activations associated with happy early stage relationships and romantic rejection, it is important to consider both “liking” (hedonic impact) and “wanting” (e.g., incentive salience) aspects of reward. That is, approach behavior and desired interaction with a person or a substance may or may not involve actual pleasurable experiences. In the context of addiction, it is often the case that a strong desire for the substance or a behavioral addiction, approach motivation and use, occurs even when the stimuli no longer provides a “high” and the reward-seeking behavior is associated with negative outcomes (e.g., the addiction is detrimental to the individual’s health, career, social relationships etc.). Those who are rejected in love still “want” the ex-partner and experience approach motivation (e.g., desiring to contact the ex-partner) even when contact with the ex may be accompanied by negative outcomes and not pleasurable (e.g., experiences of sadness and pain). A distinction between hedonic impact and incentive salience has been explored in animal studies (Berridge et al., 2009). We also found that looking at the partners face activated the right VTA while left VTA activation correlated with the attractiveness of faces in the study (Aron et al., 2005).

## Attachment

For those who stay in a relationship beyond the early stage, intense romantic phase, an important second constellation of feelings sets in, associated with attachment (Acevedo et al., 2011). In our studies of individuals who are happily in love (Fisher et al., 2003; Aron et al., 2005), we found that those in longer partnerships (8–17 months as opposed to 1–8 months) began to show activity in the ventral pallidum, associated with attachment in animal studies (Insel and Young, 2001), while continuing to show activity in the VTA and caudate nucleus associated with passionate romantic love. Thus, with time, feelings of attachment begin to accompany feelings of passionate romantic love (Fisher, 2004; Acevedo et al., 2011). Working in conjunction, these two basic neural systems for romantic love and attachment may constitute the biological foundation of human pair-bonding—and provide the context for the evolution of love addictions (Insel, 2003; Burkett and Young, 2012; Fisher, 2016).

## Evolution of Romantic Love and Attachment

It has been proposed that the neural systems associated with feelings of intense romantic love and partner attachment evolved in conjunction with the evolution of the human predisposition for pair-bonding, serving as mechanisms to stimulate mate choice and motivating individuals to remain with a mate long enough to breed and rear their offspring through infancy as a team (Fisher, 2004, 2011, 2016; Fisher et al., 2006). This hypothesis suggests that the neural systems for romantic love and attachment are survival systems with evolutionary roots (Frascella et al., 2010).

Pair-bonding is a hallmark of humanity. Data from the Demographic Yearbooks of the United Nations on 97 societies canvassed in the 1980s indicate that approximately 93.1% of women and 91.8% of men in that decade married by age 49 (Fisher, 1989, 1992). Worldwide, marriage rates have declined since then; but today 85 to 90% of men and women in the United States are projected to marry (Cherlin, 2009). Cross-culturally, most individuals are monogamous; they form a sexual and socially sanctioned partnership with one person at a time. Polygyny (many females) is permitted in 84% of human societies; but in the vast majority of these cultures, only 5 to 10% of men actually have several wives simultaneously (Van den Berghe, 1979; Frayser, 1985). Moreover, because polygyny in humans is regularly associated with rank and wealth, monogamy (i.e., pair-bonding) may have been even more prevalent in the pre-horticultural, unstratified societies of our long human hunting-gathering past (Daly and Wilson, 1983), when the neural systems for intense early stage romantic love and partner attachment most likely evolved.

Data suggest that the human predisposition for pair-bonding (often preceded by romantic attraction) also has a biological basis. The investigation of human attachment began with Bowlby (1969, 1973) and Ainsworth et al. (1978) who proposed that, to promote the survival of the young, primates have evolved an innate attachment system designed to motivate infants to seek comfort and safety from their primary caregiver, generally the mother. Since these early studies, extensive research has been done on the behaviors, feelings and neural mechanisms associated with this attachment system in adult humans and other animals (Fraley and Shaver, 2000; Eisenberger et al., 2003; Panksepp, 2003a,b; Bartels and Zeki, 2004; MacDonald and Leary, 2005; Tucker et al., 2005; Noriuchi et al., 2008). Currently, researchers believe that this biologically based attachment system remains active throughout the human life course, serving as the foundation for attachment between pair-bonded partners for the purpose of raising offspring (Hazan and Shaver, 1987; Hazan and Diamond, 2000).

Pair-bonding could have evolved at any point in hominin evolution; and with it, various love addictions (Fisher, 2016). However, two lines of data suggest that the neural circuitry for human pair-bonding may have evolved at the basal radiation of the hominin stock (Fisher, 1992, 2011, 2016), in tandem with the hominin adaptation to the woodland/savannah eco-niche some time prior to 4 million years B.P. *Ardipithecus ramidus*, currently dated at 4.4 million years B.P., displays several physical traits that have been linked with pair-bonding in many species (Lovejoy, 2009); so Lovejoy (2009) proposes that human monogamy had evolved by this time. Anthropologists have also re-measured *Australopithecus afarensis* fossils for skeletal variations; and they report that by 3.5 million years B.P. hominins exhibited roughly the same degree of sexual dimorphism in several physical traits that the sexes exhibit today. Thus, some have proposed that these hominins were “principally monogamous” (Reno et al., 2003).

The emergence of bipedalism may have been a primary factor in the evolution of the neural circuitry for hominin pair-bonding (Fisher, 1992, 2011, 2016) and the concomitant evolution of romantic love (and possibly attachment) addiction. While foraging and scavenging in the woodland/savannah eco-niche, bipedal Ardipithecine females were most likely obliged to carry infants in their arms instead of on their backs, thus needing the protection and provisioning of a mate while they transported nursing young. Meanwhile, Ardipithecine males may have had considerable difficulty protecting and providing for a harem of females in this open woodland/savannah eco-niche. But a male could defend and provision a single female with her infant as they walked near one another, within the vicinity of the larger community.

So the exigencies of bipedalism in conjunction with hominin expansion into the woodland/savannah eco-niche may have pushed Ardipithecines over the “monogamy threshold,” selecting for the neural system for attachment to a pair-bonded partner. And along with the evolution of pair-bonding and the neural system for attachment may have emerged the brain system for intense positive romantic addiction—serving to motivate males and females to focus their mating energy on a single partner and remain together long enough to trigger feelings of attachment necessary to initiate and complete their co-parenting duties of highly altricial young (Fisher, 1992, 2004, 2011, 2016).

## Human Romantic Love as a Developed Form of a Mammalian Courtship Mechanism

Considerable data suggest that the human brain system for romantic love arose from mammalian antecedents. Like humans, all birds and mammals exhibit mate preferences; they focus their courtship energy on favored potential mates and disregard or avoid others (Fisher, 2004; Fisher et al., 2006). Moreover, most of the basic traits associated with human romantic love are also characteristic of mammalian courtship attraction, including increased energy, focused attention, obsessive following, affiliative gestures, possessive mate guarding, goal-oriented behaviors and motivation to win and keep a preferred mating partner for the duration of one’s species-specific reproductive and parenting needs (Fisher et al., 2002, 2006; Fisher, 2004).

The brain system for human romantic love shows biological similarities with mammalian neural systems for courtship attraction. When a female laboratory–maintained prairie vole is mated with a male, she forms a distinct preference for him, associated with a 50% increase of dopamine in the nucleus accumbens (Gingrich et al., 2000). When a dopamine antagonist is injected into the nucleus accumbens, the female no longer prefers this partner; and when a female is injected with a dopamine agonist, she begins to prefer the conspecific who is present at the time of the infusion, even if she has not mated with this male (Wang et al., 1999; Gingrich et al., 2000). An increase in the activities of central dopamine is also associated with courtship attraction in female sheep (Fabre-Nys et al., 1997). In male rats, increased striatal dopamine release has also been shown in response to the presence of a receptive female rat (Robinson et al., 2002; Montague et al., 2004).

Because human romantic love shares many behavioral and biological characteristics with mammalian courtship attraction, it is likely that human romantic love is a developed form of this mammalian neural courtship mechanism (Fisher, 1998, 2004, 2011, 2016; Fisher et al., 2006). However, in most species courtship attraction is brief, lasting only minutes, hours, days, or weeks; while in humans, intense, early stage romantic love can last 12–18 months (Marazziti et al., 1999) or much longer (Acevedo et al., 2011). So in early hominin prehistory, activity in this mammalian neural system for courtship attraction may have become intensified and prolonged as pair-bonding evolved, eventually becoming the positive (or negative) romantic addictions experienced by men and women cross-culturally today.

## Romantic Love May Act As A Reward Replacement for Other Addictions

High quality social relationships (including romantic relationships) can be extremely beneficial to those recovering from an addiction (e.g., Hänninen and Koski-Jännes, 1999). One potential mechanism for this benefit comes from the therapeutic approach to drug addiction of reward replacement. That is, when quitting one addictive substance or behavior, the addicted individual replaces this addiction with another form of rewarding behavior, often without prompting from an outside source, such as a clinician (Donovan, 1988; Marks, 1990; DiNardo and Lemieux, 2001; Haylett et al., 2004; Alter et al., 2006). Because of this, clinicians who treat addictions are known to effectively engage patients in new reinforcers (see Bickel et al., 2014), specifically healthy replacement reinforcers such as sports activities, new hobbies and more or new social interactions (e.g., Vaillant, 1983; Salvy et al., 2009; Liu et al., 2011).

Could early stage romance provide a replacement reward for those engaged in substance abuse (or a behavioral addiction)? To explore this question, Xu et al. (2012) put 18 Chinese overnight nicotine-deprived smokers who had just fallen madly in love into a brain scanner, using fMRI. These men and women looked at side-by-side photos, one of a hand holding either a lighted cigarette (cue) or a pencil (control) and one of their newly beloved or a familiar acquaintance (non-smokers so they were not cigarette-cues). Among those who were moderately addicted to nicotine, when the cigarette cue was presented next to the image of the beloved (compared to the acquaintance), less activation was observed in regions associated with cigarette cue-reactivity. Additionally, more activation in the caudate was observed during trials that included the beloved’s pictures (compared to the acquaintance’s).

These preliminary data provide more evidence that romantic love could be considered a powerful and primordial natural addiction because it can, under some circumstances, modify brain activations associated with a more contemporary addiction, nicotine.

“Self-expansion” and “incorporation of others into one’s sense of self” may also act as reward substitutes for addictions, including love addiction.

First proposed by Aron and Aron (1986), the self-expansion model proposes that a basic human motivation is the desire to increase one’s self-concept by engaging in novel, interesting, challenging and/or other exciting pursuits in order to gain resources and perspectives that can enhance one’s self concept and capabilities (for review see Aron et al., 2013), as well as garner positive emotions and reward feelings (Aron et al., 1995, 2000; Strong and Aron, 2006). They propose that rapid self-expansion occurs during early stage romance.

This self-expansion, which is rooted in approach motivation (see Mattingly et al., 2012), may be beneficial when attempting to quit or reduce use of a substance or behavioral addiction because it offers a replacement and distracting rewarding experience. Self-expansion in the context of romantic love has been shown to attenuate perceptions of physical pain (Younger et al., 2010) via a reward mechanism (rather than distraction), which suggests that it might assist with the painful process of withdrawal after romantic rejection. Further, self-expansion may also be beneficial in the context of quitting any addiction because it facilitates self-concept change (e.g., starting to think of oneself as a writer, musician, bird watcher or whatever the self-expanding experience may be) into a new and healthier direction, and away from one’s identity as a “user” (Kellogg and Kreek, 2005). In addition to providing distraction, replacement and redirection, engaging in self-expanding (i.e., novel, interesting, and/or challenging) activities may be biologically beneficial, because any form of novelty activates the dopamine system in the brain to facilitate energy and optimism, thereby potentially providing a replacement reward.

Indeed, three studies have directly investigating self-expansion in the context of nicotine addiction, each finding quite positive results. Ex-smokers reported that significantly more self-expanding experiences had occurred directly before they successfully quit smoking than did current smokers who reported on their unsuccessful attempts to quit (Xu et al., 2010). Even among the current smokers who relapsed, the number of self-expanding experiences occurring directly before their quit attempt was significantly positively correlated with how long they were able to abstain from smoking (Xu et al., 2010). Two fMRI studies of overnight abstinent smokers suggest that self-expansion via activities with a romantic partner attenuates cigarette cue-reactivity in the brain (Xu et al., 2012, 2014). These data suggest that when smokers engage in self-expansion, they are less responsive to smoking cues.

Another cognitive phenomenon that may play a role in attenuating romantic addiction is “inclusion of the other in the self” (IOS). This occurs when representations of the self change to incorporate aspects of a romantic partner. A scale has been developed to measure this cognitive process (Aron et al., 1992). Over time the partner’s perspectives, identities, and resources become incorporated into the person’s own sense of self and the distinction between self and partner blur. For example, people transition to more use of plural pronouns like “we” and “us”(Agnew et al., 1998), and become slower at distinguishing a partner’s belongings or traits from one’s own (Aron et al., 1991; for a review, see Aron et al., 2004). This growth of the self-concept can provide positive outcomes (e.g., additional resources, positive feelings), which may be effective in a therapeutic situation. Indeed, activation of the reward system through the VTA was correlated with a lover’s IOS scores (Acevedo et al., 2011), which suggests that a moderate amount of positive identification with another person or group could be therapeutic–by boosting a positive self-image and providing a reward substitute for a substance or behavioral addiction that a person has given up.

## Implications for Treatment of Romantic Rejection and Addiction

Clinicians have a host of strategies for helping lovers and drug addicts. However, when data on romantic love and substance abuse are considered together, some approaches have a particularly strong rationale.

Perhaps most important, like giving up a drug, rejected lovers should remove all reasonable evidence of their abandoning sweetheart, such as cards, letters, songs, photos, and memorabilia, as well as avoid contact with their rejecting partner, because reminders and partner contact can act as cues that induce craving and are likely to sustain the activity of brain circuits associated with romantic passion and thus interfere with the healing process. Self-expansion research also finds that positive outcomes such as personal growth and positive emotions are possible (even likely) following a break-up if the relationship had offered few self-expanding opportunities and if the newly single person engages in rediscovery of the self (Lewandowski and Bizzoco, 2007).

Close, positive contact with a friend or friends is rewarding and may also help to replace the craving for substances or a rejecting partner, because looking at a photo of a close friend activates the nucleus accumbens, associated with reward (Acevedo et al., 2011). Looking at a photo of a close friend also activates the periaqueductal gray, associated with oxytocin receptors and the calm of attachment. This suggests that group therapies, such as Alcoholics Anonymous and other 12 step programs, are successful because these group dynamics engage the brain’s reward and attachment systems. Participating in group programs may be important for rejected lovers as well as for those addicted to substances like alcohol or those with a behavioral addiction, such as gambling.

Data suggest that rejected lovers should also stay busy to distract themselves (Thayer, 1996; Rosenthal, 2002). Physical exertion may be especially helpful as it elevates mood (Rosenthal, 2002), triggering dopamine activity in the nucleus accumbens to bestow pleasure (Kolata, 2002). Exercise also increases levels of β-Endorphin and endocannabinoids which reduces pain and increases feelings of calm and well-being (Goldfarb and Jamurtas, 1997; Dietrich and McDaniel, 2004). Also, engaging in a new form of exercise can be a self-expanding experience (see Xu et al., 2010). Because of these benefits of exercise, some psychiatrists believe that exercise (aerobic or anaerobic) can be as effective in healing depression as psychotherapy or antidepressant drugs (Rosenthal, 2002).

Self-expanding activities (e.g., hobbies, sports, spiritual experiences) can be helpful both in the context of addiction and heartbreak as they offer reward, benefits to the self-concept, and distraction. It is recommended that a person has more than one source of self-expansion in their life, thus should one no longer become available (e.g., a partner leaves), the other sources can help buffer the impact of that loss. It would also be helpful to have multiple and diverse sources of self-expansion in various domains of life (e.g., hobby, workplace, friends, family, volunteer organization, spiritual group, and academic interest etc.) and to have strong social networks to which one can turn for support in times of need (e.g., breakup, attempting to quit). It is important, however, to note that self-expansion should be pursued in a healthy manner with caution about potentially risky behaviors (e.g., seeking to fall in love with a new person immediately after the loss of a partner, picking up unhealthy habits or becoming an addict of another substance when quitting).

Similarly, it is important to remember that relationships and addictions can co-exist and influence each other and it may be especially difficult to have a strong and positive romantic relationship when issues of addiction need to be dealt with. As addiction often leads to less desire for and response to alternative rewards, it may be especially difficult for those dealing with addiction to engage in pro-relationship behaviors, and thus increase the risk of rejection. In addition, romantic rejection increases the risk of relapse, so close attention to romantic relationships during substance abuse withdrawal may be important.

Furthermore, smiling utilizes facial muscles that activate nerve pathways in the brain that can stimulate feelings of pleasure (Carter, 1998). Focusing on the positive may be effective too. A study by Lewandowski (2009) found that writing for 20 min on three consecutive days about a recent relationship break-up was beneficial when people wrote about positive feelings as opposed to when they wrote about negative feelings or wrote without expressing any feelings. Perhaps most important, time attenuates the attachment system. In our study of rejected men and women, the greater the number of days since rejection, the less the activity in a brain region (the ventral pallidum) associated with feelings of attachment (Fisher et al., 2010).

As disappointed lovers use strategies originally developed to quit a substance addiction, their love addiction is likely to eventually subside.

## Conclusion

Researchers have long discussed whether the compulsive pursuit of non-substance rewards, such as uncontrolled gambling, eating, sex, exercise, Internet use, compulsive buying disorder and other obsessive behavioral syndromes can be classified as addictions (Frascella et al., 2010; Rosenberg and Feder, 2014). All can lead to salience, obsession, tolerance, emotional, and physical dependence, withdrawals, relapse and other traits common to substance abuse. Moreover, several of these non-substance rewards have been shown to produce specific activity in dopamine pathways of the reward system similar to drugs of abuse (see Frascella et al., 2010; see Rosenberg and Feder, 2014). This suggests that uncontrolled use of these non-substances can be considered addictions. Romantic love is likely to be a similar addiction, with one exception. Unlike other addictions (that aﬄict only a percentage of the population), some form of love addiction is likely to occur to almost every human being that lives now and in our human past; few avoid the pain of romantic rejection either.

Romantic love appears to be a natural addiction, “a normal altered state” experienced by almost all humans (Frascella et al., 2010, p. 295) that evolved during human evolution to motivate our ancestors to focus their mating energy on a specific partner, thereby conserving mating time and energy, initiating reproduction, triggering feelings of attachment and subsequent mutual parenting, and assuring the future of their mutual DNA (Fisher, 2004, 2011, 2016; Fisher et al., 2006). Romantic love may be a positive addiction when the relationship is reciprocated, non-toxic and appropriate; but a harmful, negative addiction when unreciprocated, toxic, inappropriate and/or formally rejected.

To alleviate the negative symptoms of love addiction, addicted lovers are advised to remove the cues that fan their ardor, follow some advisories of a 12-step program, build new daily habits, meet new people, take up new interests, find the appropriate medication and/or therapist, and wait out the days and nights of intrusive thinking and craving, because feelings of attachment to a former romantic partner decrease over time (Fisher et al., 2010). Moreover, therapies that increase self expansion and incorporate new individuals into one’s sense of self may also be useful in alleviated love addiction. Self expansion approaches may help drug and other negative addiction therapies, also.

If the public and the therapeutic, medical and legal communities come to understand that passionate early stage romantic love is an evolved drive (Fisher, 2004) and a natural addiction (Frascella et al., 2010) that can have profound social, economic, psychological, and genetic consequences (both beneficial and adverse), clinicians and researchers might develop more effective procedures for dealing with this powerful and primordial neural mechanism for mate preference and initial partner attachment, romantic love.

## Author Contributions

HF wrote half the text based on her ideas and data from previous studies and edited the final version. XX wrote twenty percent of the text based on her ideas and data from previous studies. AA contributed to the text based on his ideas and previous studies. LB wrote thirty percent of the text based on her ideas and data from previous studies and edited the final version.

## Conflict of Interest Statement

The authors declare that the research was conducted in the absence of any commercial or financial relationships that could be construed as a potential conflict of interest.
